# Outcomes of Early Extubation Following Fontan Surgery in Children: A Systematic Review and Meta-Analysis

**DOI:** 10.7759/cureus.98561

**Published:** 2025-12-06

**Authors:** Ahmed M Omran, Mohammed M Elabd

**Affiliations:** 1 Pediatric Intensive Care Unit, King Fahad Medical City, Riyadh, SAU

**Keywords:** fontan procedure, length of stay in icu, pediatric cardiac surgery outcomes, reintubation, systematic review and meta analysis

## Abstract

Children with single-ventricle physiology undergo the Fontan procedure but remain at risk of postoperative complications. Whether early extubation (EE) is safe and beneficial in this population is uncertain.

The objective of this study was to assess the safety and effectiveness of EE compared with delayed extubation after Fontan surgery in children. We conducted a Preferred Reporting Items for Systematic Reviews and Meta-Analyses (PRISMA)-guided systematic review and meta-analysis registered in the International Prospective Register of Systematic Reviews (PROSPERO) (CRD420251083062). We searched PubMed, Scopus, and Web of Science from January 1, 2005, to June 2025. Eligible studies compared EE (within six to 12 hours) versus delayed extubation in patients aged zero to 18 years undergoing the Fontan procedure.

Outcomes included reintubation (primary), ICU and hospital length of stay, ventilation time, mortality, and postoperative complications. Risk of bias was assessed with Risk Of Bias In Non-randomized Studies of Interventions (ROBINS-I); certainty of evidence was rated using Grading of Recommendations, Assessment, Development, and Evaluation (GRADE).

Four retrospective cohort studies met the inclusion criteria. EE was not associated with a higher risk of reintubation. EE was associated with a shorter ICU stay. There were no significant differences in hospital length of stay or pleural effusion. Mortality did not differ significantly between groups (pooled RR 0.30, 95% CI 0.08-1.18; p = 0.09). Across outcomes, statistical heterogeneity was low. Overall certainty of evidence was low, primarily due to observational designs and imprecision.

In pediatric Fontan patients, EE appears safe and is associated with a shorter ICU stay without increased reintubation or mortality. These findings support EE within structured fast-track pathways for appropriately selected patients, while highlighting the need for prospective, higher-quality studies.

## Introduction and background

Children with single-ventricle physiology undergo staged palliation culminating in the Fontan procedure, which reroutes systemic venous return directly to the pulmonary arteries. Although surgical outcomes have improved, patients with a Fontan circulation remain at risk for unique short- and long-term complications that demand careful perioperative management (Rychik et al.) [1]. These physiologic complexities increase the importance of optimizing immediate postoperative care to improve early outcomes and resource use, highlighting the need for Fontan-specific evidence synthesis to guide best practices (Rao et al.) [2]. Positive-pressure mechanical ventilation increases intrathoracic pressure and may elevate pulmonary vascular resistance, thereby reducing systemic venous return and cardiac output, effects that can be particularly detrimental in the Fontan circulation, where pulmonary blood flow is passive (Corp et al.) [3]. Minimizing invasive ventilation time and avoiding sustained positive pressures are therefore physiologically plausible strategies to preserve Fontan hemodynamics (Charla et al.) [4]. Early extubation (EE), commonly defined as planned extubation within the first six to 12 hours after surgery or even immediately in the operating room, is a central component of fast-track cardiac anesthesia (Tham et al.) [5]. However, despite these physiologic rationales and some observational data, the safety and impact of EE specifically in the Fontan population remain unclear. Therefore, this study aims to systematically synthesize Fontan-specific evidence to clarify the effects of early versus delayed extubation on reintubation, mortality, ICU/hospital stay, and other postoperative outcomes.

In broader pediatric cardiac surgery cohorts, EE has been associated with reduced ICU and hospital length of stay, lower sedative use, earlier detection of complications, and, in some reports, comparable or lower reintubation rates. These benefits are likely mediated through reduced ventilator-associated physiological disturbances, decreased sedation-related risks, and faster mobilization (Garg et al.) [6]. Several observational studies specifically in the Fontan population report favorable early hemodynamics and shorter ICU interventions after EE, but findings are heterogeneous and mostly retrospective. Representative cohorts (Ovroutski et al.) [7], (Kawaguchi et al.) [8], (Ono et al.) [9], and (Kintrup et al.) [10] suggest potential benefits but are limited by study design, small sample sizes, and variable extubation criteria. Consequently, a quantitative synthesis is needed to clarify safety (reintubation, mortality) and the effect on resource-related outcomes (ICU/hospital stay). Given the physiological rationale and accumulating but inconsistent observational data, we performed a systematic review and meta-analysis of studies comparing early versus delayed extubation after Fontan surgery in children. Our primary objective was to evaluate reintubation risk; secondary objectives included ICU and hospital length of stay, ventilation time, postoperative complications (e.g., pleural effusion), and mortality, and to assess the certainty of evidence using Grading of Recommendations, Assessment, Development, and Evaluation (GRADE).

## Review

Methods

Protocol and Registration

This systematic review and meta-analysis were conducted in accordance with the Preferred Reporting Items for Systematic Reviews and Meta-Analyses (PRISMA) 2020 guidelines. The protocol was prospectively registered in the International Prospective Register of Systematic Reviews (PROSPERO; registration ID: CRD420251083062).

Eligibility Criteria

Population: Pediatric patients (age zero to 18 years) undergoing the Fontan procedure (any surgical technique: lateral-tunnel, extracardiac total cavopulmonary connection (TCPC), etc.).

Intervention: EE was defined a priori as planned removal of the endotracheal tube within six to 12 hours after completion of the index surgery (including extubation in the operating room or early in the ICU as defined by each study).

Comparison: Delayed extubation (extubation later than the EE window as defined by each study).

Outcomes: Primary outcome was reintubation (within the same admission). Secondary outcomes included ICU length of stay (days), hospital length of stay (days), mechanical ventilation time (hours), postoperative complications (e.g., clinically significant pleural effusion requiring drainage), and in-hospital mortality.

Study designs: Randomized controlled trials (RCTs) or controlled observational cohort studies (prospective or retrospective) that directly compared EE versus delayed extubation. Case reports, case series without a comparator group, narrative reviews, editorials, and conference abstracts without full text were excluded.

Timeframe and language: Studies published from January 1, 2005, to June 30, 2025, in English were considered.

Information Sources and Search Strategy

We performed comprehensive searches in PubMed (Medical Literature Analysis and Retrieval System Online (MEDLINE)), Scopus, and Web of Science. The last search date was June 30, 2025. We also screened reference lists of included studies and recent reviews for additional eligible reports and searched trial registries when appropriate. Full, database-specific strategies for PubMed, Scopus, and Web of Science are provided in Appendix 1.

Study Selection

All retrieved records were imported into Rayyan Qatar Computing Research Institute (QCRI), Doha, Qatar, or another systematic-review tool for deduplication and blinded screening.

Two reviewers (Dr. Ahmed Omran and Dr. Mohamed Elabd) independently screened titles and abstracts for eligibility. Full texts of potentially eligible articles were retrieved and independently assessed by the same two reviewers. Discrepancies were resolved by discussion and, if necessary, adjudication by a third reviewer.

Data Extraction and Management

Two reviewers independently extracted data using a pilot-tested standardized extraction form. Extracted items included author, year, country, study design, sample size (EE and delayed groups), patient age (mean or median), Fontan type, extubation timing/definition, baseline characteristics and important confounders (e.g., cardiopulmonary bypass time, complexity scores), outcomes of interest (event counts for dichotomous outcomes; means/SD or medians/IQR for continuous outcomes), and follow-up period. When only medians and interquartile ranges (or ranges) were reported, we converted these to mean ± SD using the methods of Wan et al. [11]. For unclear or missing outcome data, we attempted to contact the corresponding authors by email (two attempts spaced one week apart), and the study was included/excluded according to available data and predefined rules. Duplicate or overlapping cohorts (multiple reports from the same center/population) were identified; when overlap was detected, we included the most complete or recent dataset.

Data items for extraction (summary): Study ID; design; sample sizes (EE / delayed); patient age and sex distribution; Fontan type; extubation protocol (timing and criteria); baseline risk factors; outcomes (reintubation events, ICU length of stay, hospital length of stay, ventilation time, complications, deaths); risk-of-bias judgments; notes on missing data/contact attempts.

Risk of Bias Assessment

For non-randomized studies, we used the Risk Of Bias In Non-randomized Studies of Interventions (ROBINS-I) tool. Two reviewers independently assessed each included study across the ROBINS-I domains (confounding, selection of participants, classification of interventions, deviations from intended interventions, missing data, measurement of outcomes, and selection of reported results) and assigned domain-level and overall risk-of-bias judgments (low, moderate, serious, or critical). Disagreements were resolved by consensus and a third reviewer if required.

For non-randomized studies, we used the ROBINS-I tool because it is specifically designed to assess risk of bias in observational and quasi-experimental studies. ROBINS-I evaluates seven domains, including confounding, selection of participants, classification of interventions, deviations from intended interventions, missing data, measurement of outcomes, and selection of reported results. This comprehensive approach allows for a structured and transparent assessment of bias, which is crucial when synthesizing evidence from non-randomized studies.

Data Synthesis and Statistical Analysis

All meta-analyses were performed using Review Manager (RevMan 5.4, The Cochrane Collaboration, London, UK) for primary pooling, with supplementary and sensitivity analyses conducted in R (R Foundation for Statistical Computing, Vienna, Austria) (packages meta and metafor) when advanced methods were required. For dichotomous outcomes (reintubation, complications, mortality), pooled risk ratios (RRs) with 95% confidence intervals (CIs) were calculated, while continuous outcomes (ICU length of stay, hospital length of stay, ventilation time) were analyzed using mean differences (MDs) and 95% CIs when reported on the same scale; standardized mean differences (SMDs) were planned for outcomes reported on differing scales. A random-effects model was applied as the primary analytic approach to account for expected clinical heterogeneity across centers and extubation protocols. The DerSimonian-Laird (DL) estimator was used in RevMan analyses, and Hartung-Knapp-Sidik-Jonkman (HKSJ) adjustments were performed in R for sensitivity analyses to yield more conservative CIs, particularly given the small number of included studies. For rare binary outcomes such as mortality, additional sensitivity analyses were performed using the Mantel-Haenszel method with a 0.5 continuity correction and the Peto method when event rates were extremely low, and treatment effects were small and balanced. Studies with zero events in both arms were excluded from pooled estimates but described qualitatively; generalized linear mixed models (GLMM) were also considered for rare events in R. When studies reported medians and IQRs (or ranges) instead of means and SDs, these values were converted using the approach described by Wan et al. [11], with sensitivity analyses conducted excluding converted data. Statistical heterogeneity was evaluated using the I² statistic and Cochran’s Q (Chi²) test, with I² interpreted as low (0-25%), moderate (26-50%), and high (>50%), and sources of heterogeneity explored through prespecified subgroup and sensitivity analyses [12]. Additional sensitivity analyses included exclusion of studies at serious or critical risk of bias (ROBINS-I) [13], leave-one-out influence analyses, and comparison of DL versus HKSJ estimators. Subgroup analyses were interpreted cautiously and performed only when at least two studies contributed data to each subgroup, while meta-regression was not planned due to limited study numbers. Publication bias was assessed using funnel plots and Egger’s test only if ten or more studies were pooled for a given outcome; given the small sample size, the potential for publication bias was instead discussed qualitatively.

Certainty of Evidence

We assessed the certainty of evidence for key outcomes using the GRADE approach (high, moderate, low, very low) across domains of risk of bias, inconsistency, indirectness, imprecision, and publication bias [14].

Handling Missing Data and Author Contact

For studies with missing critical data (e.g., means/SDs or event counts), we contacted corresponding authors via e-mail (two attempts, one week apart). If no response was received, we used available data (e.g., medians converted to means) or excluded the outcome from pooled analysis if conversion was not possible.

Software

Primary meta-analyses and forest plots were generated using RevMan 5.4. Sensitivity analyses and Hartung-Knapp adjustments were performed in R (version ≥4.0) using the meta and metafor packages.

Figures and tables were prepared in Microsoft Word/Excel (Microsoft Corporation, Redmond, Washington, United States) and converted to the journal’s required formats.

Results

**Figure 1:**
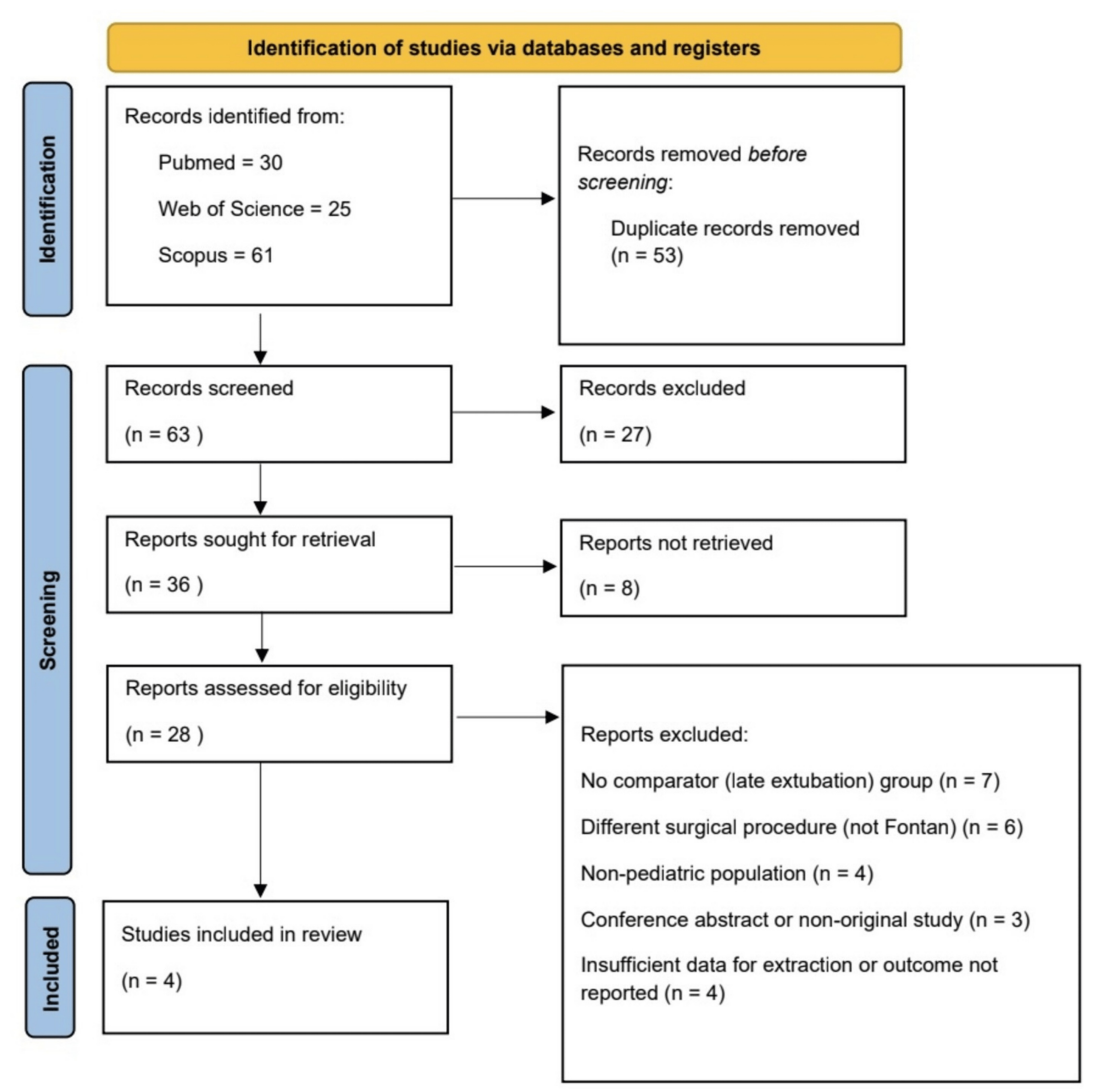
The PRISMA 2020 flow diagram PRISMA: Preferred Reporting Items for Systematic Reviews and Meta-Analyses

After screening and eligibility assessment according to PRISMA 2020, four studies met the inclusion criteria and were included in the qualitative and quantitative synthesis (Figure 1).

The database search yielded 116 records. After removing duplicates, 63 records were screened by title and abstract. Following title and abstract screening, 36 reports were sought for retrieval, and 28 full-text articles were assessed for eligibility. Twenty-four full-text reports were excluded for reasons detailed in the PRISMA flow diagram. Ultimately, four studies met the inclusion criteria and were included in the review. The study selection process is illustrated in the PRISMA 2020 flow diagram (Figure 1).

The included studies (Ovroutski et al.) [7], (Kawaguchi et al.) [8], (Ono et al.) [9], and (Kintrup et al.) [10] collectively enrolled pediatric patients undergoing the Fontan procedure, with sample sizes ranging from 56 to 458 participants. The mean age across studies ranged from 2.6 to 6.5 years. Three studies were conducted in Europe (Germany) and one in Canada. All studies compared EE (within six to 12 hours, including extubation in the operating room in some protocols) versus delayed extubation. Baseline study characteristics are summarized in Table 1*.*

**Table 1:** Summarizes baseline study characteristics N: sample size; EE: early extubation; LE: late extubation; TCPC: total cavopulmonary connection; ICU: intensive care unit

Study	Year	Country	Design	N (EE / LE)	Extubation Cut-off	Fontan Type	Outcomes Reported
Ovroutski et al. [7]	2018	Germany	Retrospective cohort	59/152	12 hours	Extracardiac TCPC	ICU stay, hospital stay, mortality
Ono et al. [9]	2019	Germany	Retrospective cohort	257/201	6 hours	Extracardiac	Reintubation, ICU stay, ventilation time
Kintrup et al. [10]	2019	Germany	Retrospective cohort	60/54	6 hours	Lateral tunnel/extracardiac	Reintubation, ICU stay, complications
Kawaguchi et al. [8]	2016	Canada	Retrospective cohort	88/39	6 hours	Extracardiac	Reintubation, ventilation time, ICU stay, fluid balance

Risk of Bias 

The risk of bias for the four included studies was assessed using the ROBINS-I tool [13]. Each study was evaluated across seven domains: confounding, selection of participants, classification of interventions, deviations from intended interventions, missing data, measurement of outcomes, and selection of reported results.

**Figure 2:**
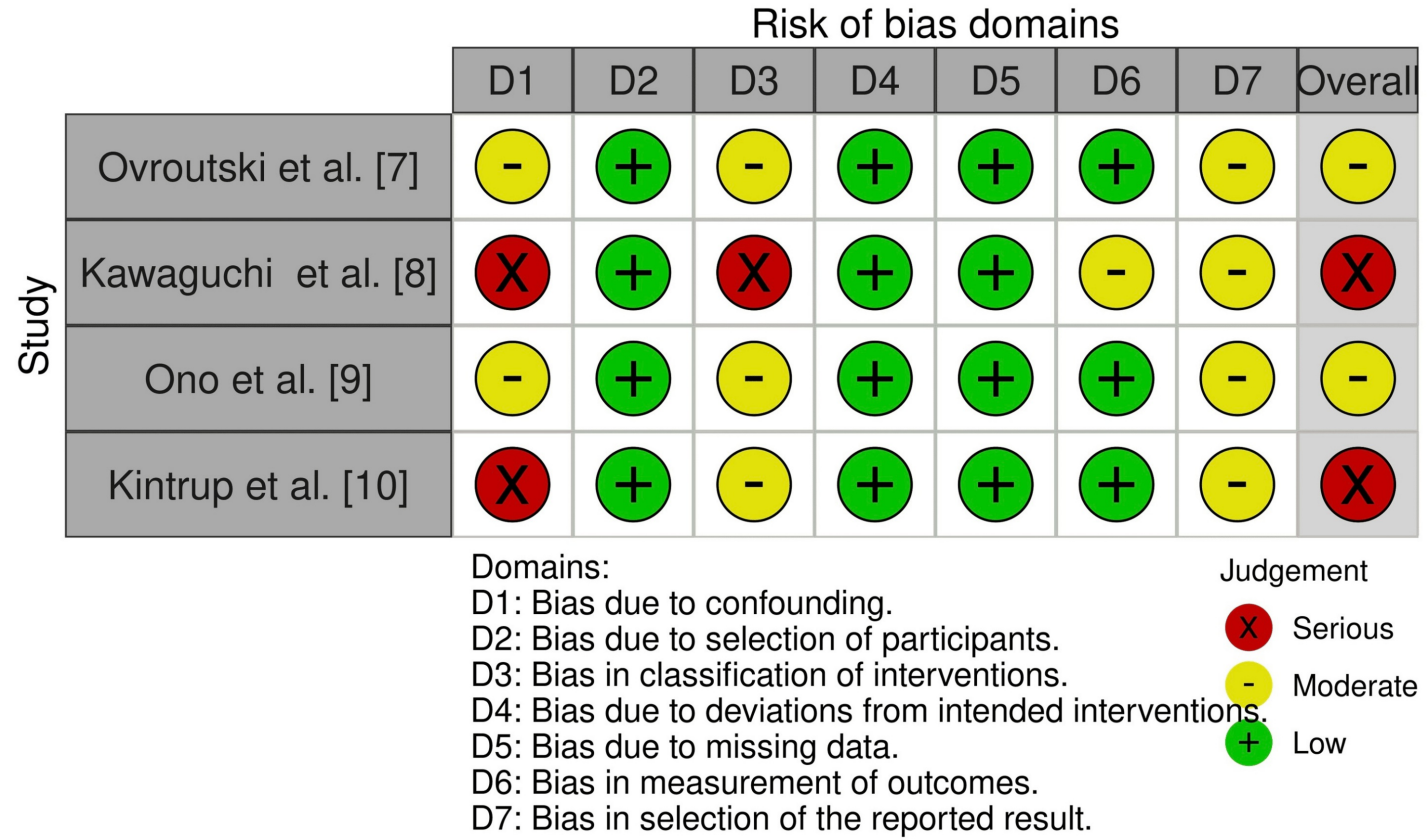
ROBINS-I risk of bias summary table for included studies ROBINS-I: Risk Of Bias In Non-randomized Studies of Interventions [7-10]

**Figure 3:**
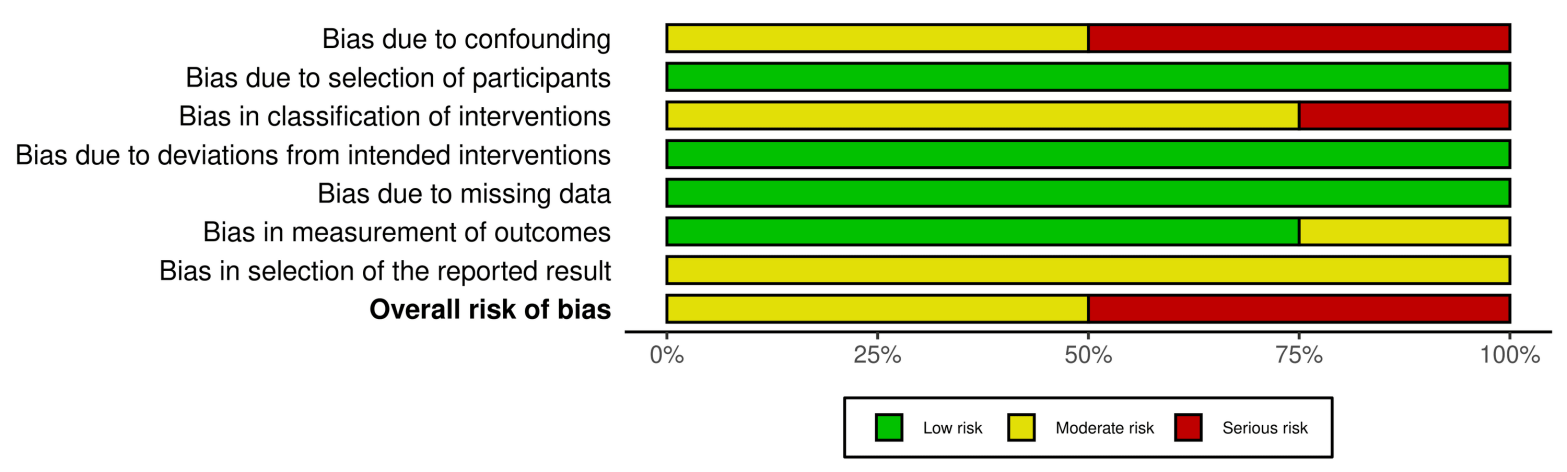
ROBINS-I risk of bias traffic light plot ROBINS-I: Risk Of Bias In Non-randomized Studies of Interventions

All included studies were retrospective cohort designs, which are inherently at higher risk of bias, particularly in the domains of confounding and classification of interventions. Two studies, Kintrup et al. [10] and Kawaguchi et al. [8], were judged to have a serious overall risk of bias, while Ovroutski et al. [7] and Ono et al. [9] were rated as having a moderate overall risk of bias, primarily due to limited control of confounding variables and incomplete reporting of intervention classification. A summary and traffic light plot of the risk of bias assessments are presented in Figures 2, 3, respectively.

*Meta-Analysis Results* 

**Figure 4:**
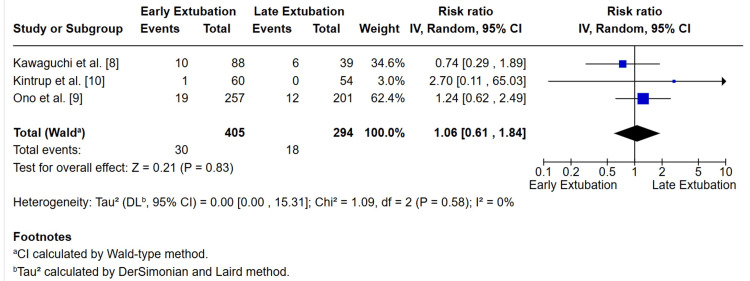
Forest plot of reintubation rates comparing early versus delayed extubation after Fontan surgery Data from three included studies were pooled using a random-effects model. No significant difference was observed between groups (Z = 0.21, P = 0.83). Heterogeneity was low (Tau² = 0.00 (95% CI: 0.00–15.31); Chi² = 1.09, df = 2, P = 0.58; I² = 0%). [8-10]

Reintubation was reported in three studies, Kawaguchi et al. [8], Ono et al. [9], and Kintrup et al. [10], including a total of 699 patients (405 in the EE group and 294 in the delayed group). The pooled analysis demonstrated no significant difference in the risk of reintubation between early and delayed extubation (RR = 1.06; 95% CI 0.61-1.84; p = 0.83). Heterogeneity was low (I² = 0%). Although the study by Kintrup et al. [10] contributed limited weight due to the small number of events, the overall findings support the safety of EE protocols in appropriately selected pediatric Fontan patients (Figure 4). 

*ICU Length of Stay* 

**Figure 5:**
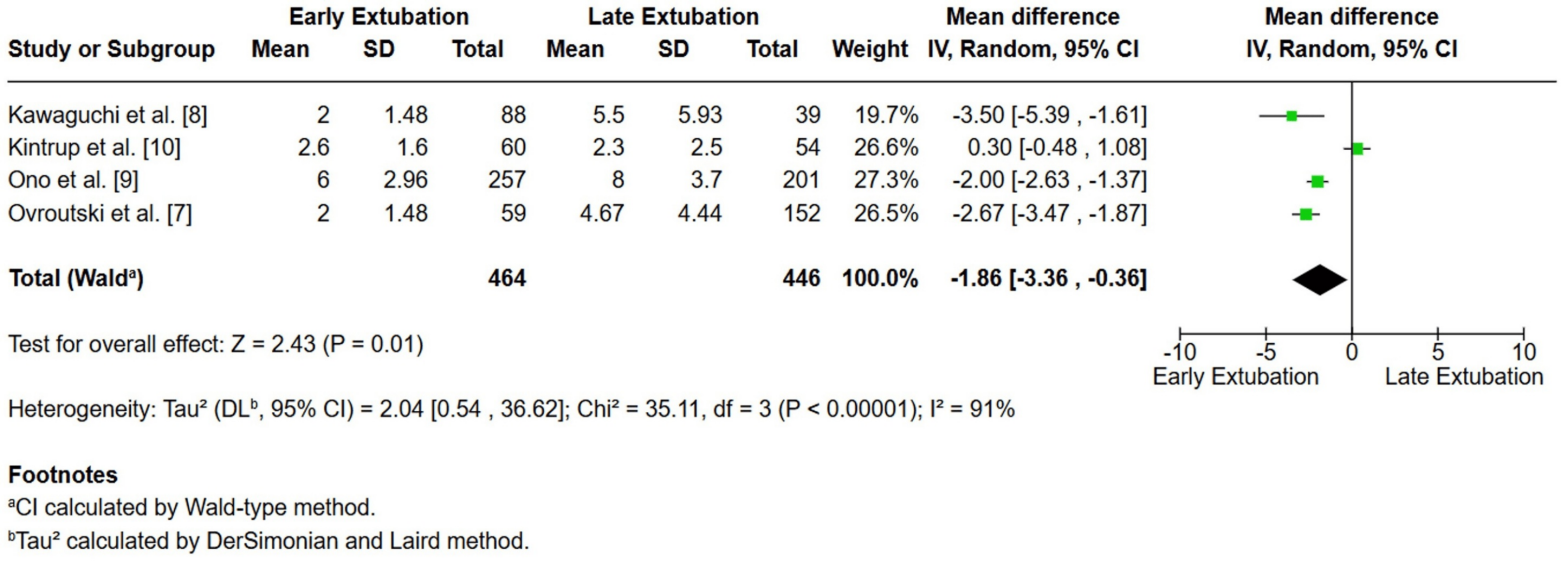
Forest plot comparing ICU length of stay between early and late extubation following Fontan surgery in pediatric patients Early extubation was associated with a significantly shorter ICU stay (mean difference = –1.86 days, 95% CI –3.36 to –0.36; P = 0.01). Considerable heterogeneity was observed across studies (I² = 91%), reflecting differences in institutional protocols, patient populations, and perioperative management. [7-10]

Four studies, Ovroutski et al. [7], Kawaguchi et al. [8], Ono et al. [9], and Kintrup et al. [10], reported ICU length of stay. In total, 910 patients were analyzed (464 early vs. 446 delayed). EE was associated with a significantly shorter ICU stay (MD −1.86 days; 95% CI −3.36 to −0.36; p = 0.01). However, substantial heterogeneity was observed (I² = 91%; p < 0.00001), largely driven by the neutral findings (Figure 5) [10]. 

*Hospital Length of Stay* 

**Figure 6:**
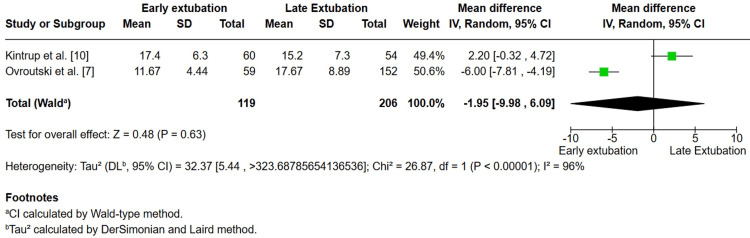
Forest plot comparing hospital length of stay between early and late extubation following Fontan surgery Pooled results from two studies (Kintrup et al. and Ovroutski et al.) showed no significant difference (MD = –1.95 days, 95% CI –9.98 to 6.09; P = 0.63). Considerable heterogeneity was observed (I² = 96%). [7,10]

Two studies, Kintrup et al. [10] and Ovroutski et al. [7], reported hospital length of stay. The pooled analysis of 325 patients demonstrated no significant difference between early and delayed extubation (MD −1.95 days; 95% CI −9.98 to 6.09; p = 0.63). However, heterogeneity was extremely high (I² = 96%), reflecting conflicting results between studies: Kintrup et al. [10] observed a slightly longer stay in the early group, whereas Ovroutski et al. [7] reported a substantially shorter stay (Figure 6).

*Mortality* 

**Figure 7:**
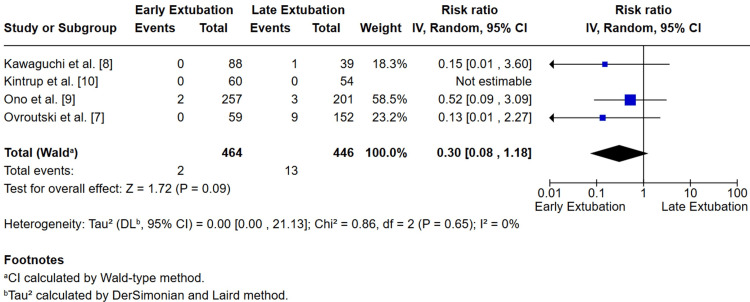
Forest plot of mortality comparing early versus late extubation after Fontan surgery The pooled estimate of four studies showed a non-significant trend toward lower mortality with early extubation (RR = 0.30, 95% CI 0.08–1.18; P = 0.09; I² = 0%). [7-10]

All four studies reported postoperative mortality. Across 910 patients, mortality occurred in two of 464 patients (0.4%) in the early group versus 13 of 446 (2.9%) in the delayed group. The pooled analysis demonstrated a non-significant trend toward lower mortality with EE (RR 0.30; 95% CI 0.08-1.18; p = 0.09). No heterogeneity was detected (I² = 0%) (Figure 7). 

*GRADE Summary of Findings* 

The certainty of evidence was rated low across all outcomes, primarily due to observational study design, imprecision, and inconsistency (Table 2). 

**Table 2:** GRADE summary of findings GRADE: Grading of Recommendations, Assessment, Development, and Evaluation

Outcome	No. of Participants (studies)	Relative Effect (95% CI)	Absolute Effect	Certainty of Evidence (GRADE)	Comments
Reintubation	699 (3 studies)	RR 1.06 (0.61–1.84)	7.4% vs 6.1%	⬤⬤◯◯ Low	Wide CI, very few events; all observational studies
ICU length of stay	910 (4 studies)	MD −1.86 days (−3.36 to −0.36)	Shorter by ~2 days	⬤⬤◯◯ Low	Significant reduction, but very high heterogeneity (I² = 91%)
Hospital stays	325 (2 studies)	MD −1.95 days (−9.98 to 6.09)	No difference	⬤⬤◯◯ Low	Conflicting results; extreme heterogeneity (I² = 96%)
Mortality	910 (4 studies)	RR 0.30 (0.08–1.18)	0.4% vs 2.9%	⬤⬤◯◯ Low	Trend toward benefit, but underpowered with very few deaths

*Subgroup and Sensitivity Analyses* 

Due to the small number of included studies (n = 4), subgroup and sensitivity analyses (e.g., stratification by extubation cut-off, geography, or Fontan type) were not feasible. However, the direction of effect was largely consistent across studies.

Publication Bias

Funnel plot analysis and Egger’s test were not performed because fewer than ten studies were included, which limits the reliability of these assessments.

Discussion 

To our knowledge, this is the first meta-analysis focused exclusively on Fontan patients, adding new evidence to the debate about the safety and benefits of EE in this population. Four observational studies, including 910 pediatric patients undergoing the Fontan procedure, were analyzed. EE was not associated with a higher risk of reintubation and was linked to a shorter ICU stay. Mortality was low overall but tended to be lower in the EE group, although the difference was not statistically significant. Hospital stays showed inconsistent results, likely reflecting variability in institutional protocols and patient selection.

Evidence From Randomized Controlled Trials

Although randomized trials in this field are limited, three studies in children provide important insights. The first RCT by Preisman et al. [15] included 100 pediatric patients. EE in the operating room significantly reduced both ICU and hospital length of stay, while reintubation and mortality rates were similar to those in the delayed extubation group, findings consistent with the trends observed in our meta-analysis, although hospital stay did not show a clear benefit.

A decade later, Ferreira et al. [16] again evaluated EE in children after congenital heart surgery. They confirmed a shorter ICU stay in the early group, with no increase in reintubation or mortality, supporting our conclusion that EE is safe and may shorten ICU recovery time.

Naguib et al. conducted the most recent RCT, including 30 infants undergoing cardiac surgery. In the EE group, there were no reintubations and no deaths, supporting our finding that EE does not increase risk [17]. They also observed smoother ICU recovery and, importantly, reported no adverse neurodevelopmental effects during follow-up, providing further reassurance about the safety of this approach in very young patients.

Taken together, these RCTs support the safety and potential benefits of EE, although their number remains small and only a few directly address the Fontan population.

Evidence From Observational Studies

Findings from observational cohorts also align with our results. Barash et al. studied 197 pediatric patients, nearly half under three years old, and achieved a 72% success rate of EE in the operating room, with only a 4% reintubation rate. Mortality, ICU stay, and hospital stay were all lower in the early group [18]. These findings are consistent with our analysis showing no excess risk of reintubation and a trend toward reduced mortality, although the reduction did not reach statistical significance in our pooled data.

Morales et al. examined outcomes in children extubated in the operating room compared with those extubated later. EE was associated with a shorter ICU stay (3 vs. 4.7 days), a shorter hospital stay (8.6 vs. 11.3 days), and reduced use of inotropes and chest drains. Importantly, there were no reintubations in the early group [19]. These findings reinforce our results that EE shortens ICU recovery, although, unlike Morales et al., we did not observe a consistent reduction in hospital stay across studies [19].

More recently, Figueroa et al. reported data from a tertiary center in Mexico City, observing a reintubation rate of 7.5% with EE versus 16.9% with delayed extubation (p = 0.001). Both ICU and hospital stays were shorter in the early group [20]. This aligns with our findings of a lower reintubation risk and shorter ICU recovery, although our meta-analysis showed greater variability in hospital stay results between centers.

Substantial heterogeneity observed in our pooled analysis appears to be driven by variation in institutional protocols, perioperative management strategies, and postoperative care pathways across centers. Such differences are well-recognized determinants of extubation outcomes and ICU recovery in pediatric cardiac surgery populations [5].

Overall, despite their retrospective design and potential biases, these observational studies consistently demonstrate that EE in children is feasible, safe, and often associated with improved short-term outcomes. Their results parallel our main findings, showing no increase in reintubation or mortality and a potential reduction in ICU stay, while highlighting that hospital stay benefits may vary depending on institutional practices.

Reviews and Meta-Analyses

Several systematic reviews have addressed this topic. Alghamdi et al. concluded that EE appeared safe but that available studies were methodologically weak, preventing firm guideline recommendations [21]. More recent narrative reviews, such as Taksande et al., highlighted the benefits of EE, including reduced ventilator-related complications, less sedation, and faster recovery [22]. Tham and Lim et al. emphasized the importance of careful patient selection and strong ICU support, noting that extubation failure can increase morbidity [5]. The most recent review by Tapioca et al. also supported EE and stressed the need for high-quality RCTs [23]. Together, these reviews reinforce our findings but also underline the limitations of the current evidence base.

Clinical Implications

Our results, based on observational data and limited randomized evidence, suggest that EE is generally safe in selected pediatric Fontan patients, with careful patient selection and structured protocols. Potential benefits include shorter ICU stays and possible cost savings, without evidence of increased reintubation or mortality risk. However, this strategy may not be appropriate for all patients, particularly those with unstable hemodynamics or significant comorbidities, and further high-quality studies are needed to confirm these findings.

Strengths and Limitations

This review represents the most comprehensive synthesis to date focused on Fontan patients, adhering to PRISMA standards and evaluating certainty using GRADE. Limitations include the small number of available studies, reliance on retrospective designs, variability in definitions of EE, and inconsistent reporting of secondary outcomes. Publication bias could not be assessed due to the limited number of studies (<10), and selective reporting cannot be ruled out. Furthermore, the few available RCTs were not exclusively conducted in Fontan patients, reducing their direct applicability.

Future Research Directions

Future studies should focus on large multicenter RCTs in the Fontan population to confirm safety and determine the optimal timing of extubation. Development of prediction tools for patient selection and evaluation of long-term outcomes, such as neurodevelopment and quality of life, is also needed. Standardized reporting of complications and resource utilization would further strengthen the evidence base.

## Conclusions

In summary, EE following Fontan surgery appears to be a generally safe and feasible strategy in carefully selected pediatric patients. It is associated with a shorter ICU stay without clear evidence of increased risk of reintubation or mortality. Although supportive evidence exists from observational studies and a limited number of RCTs, larger, multicenter trials specifically focused on the Fontan population are needed to confirm these findings and guide standardized clinical practice.

## Appendices

Appendix 1

PubMed = 30 (Advanced search)

((("Airway Extubation"[Mesh] OR "early extubation"[tw] OR "fast-track extubation"[tw] OR "early weaning"[tw] OR "extubation time"[tw] OR "extubation timing"[tw] OR "late extubation"[tw] OR "rapid extubation"[tw] OR "fast extubation"[tw])) AND (("Outcome Assessment, Health Care"[Mesh] OR outcome*[tw] OR "postoperative complications"[tw] OR mortality[tw] OR morbidity[tw] OR "ICU stay"[tw] OR "length of stay"[tw] OR "mechanical ventilation duration"[tw] OR "hospital stay"[tw] OR reintubation[tw] OR "reintubation rate*"[tw] OR "survival rate"[tw] OR "clinical outcome*"[tw] OR "respiratory outcome*"[tw]))) AND (("Fontan Procedure"[Mesh] OR "Norwood Procedures"[Mesh] OR "Fontan operation"[tw] OR "Total cavopulmonary connection"[tw] OR "extracardiac Fontan"[tw] OR "Lateral tunnel Fontan"[tw] OR "Single ventricle palliation"[tw] OR "Univentricular heart surgery"[tw] OR "Stage III palliation"[tw] OR "Fontan completion"[tw] OR "Single-ventricle physiology"[tw]))

Web of Science = 25

TS=("Airway Extubation" OR "early extubation" OR "fast-track extubation" OR "early weaning" OR "extubation time" OR "extubation timing" OR "late extubation" OR "rapid extubation" OR "fast extubation") AND TS=("Outcome Assessment" OR outcome* OR "postoperative complications" OR mortality OR morbidity OR "ICU stay" OR "length of stay" OR "mechanical ventilation duration" OR "hospital stay" OR reintubation OR "reintubation rate*" OR "survival rate" OR "clinical outcome*" OR "respiratory outcome*") AND TS=("Fontan Procedure" OR "Norwood Procedures" OR "Fontan operation" OR "Total cavopulmonary connection" OR "extracardiac Fontan" OR "Lateral tunnel Fontan" OR "Single ventricle palliation" OR "Univentricular heart surgery" OR "Stage III palliation" OR "Fontan completion" OR "Single-ventricle physiology") AND PY=(2005-2025)

Scopus = 61  (Advanced search)

TITLE-ABS-KEY ( "Airway Extubation" OR "early extubation" OR "fast-track extubation" OR "early weaning" OR "extubation time" OR "extubation timing" OR "late extubation" OR "rapid extubation" OR "fast extubation" ) AND TITLE-ABS-KEY ( "Outcome Assessment" OR outcome* OR "postoperative complications" OR mortality OR morbidity OR "ICU stay" OR "length of stay" OR "mechanical ventilation duration" OR "hospital stay" OR reintubation OR "reintubation rate*" OR "survival rate" OR "clinical outcome*" OR "respiratory outcome*" ) AND TITLE-ABS-KEY ( "Fontan Procedure" OR "Norwood Procedures" OR "Fontan operation" OR "Total cavopulmonary connection" OR "extracardiac Fontan" OR "Lateral tunnel Fontan" OR "Single ventricle palliation" OR "Univentricular heart surgery" OR "Stage III palliation" OR "Fontan completion" OR "Single-ventricle physiology" )
